# Potential and weak links in the management of tuberculosis by Pakistani private pharmacy staff

**DOI:** 10.3389/fpubh.2023.983997

**Published:** 2023-03-09

**Authors:** Fatima Balquis, Muhammad Farhan Sohail, Huma Hamid, Waseem Ullah, Amer Hayat Khan, Gul Shahnaz

**Affiliations:** ^1^Department of Pharmacy, Quaid-i-Azam University, Islamabad, Pakistan; ^2^Department of Pharmacy Practice, Shifa College of Pharmaceutical Sciences, Shifa Tameer-e-Millat University, Islamabad, Pakistan; ^3^Riphah Institute of Pharmaceutical Sciences, Riphah International University–Lahore Campus, Lahore, Pakistan; ^4^Riphah Institute of Pharmaceutical Sciences, Riphah International University–Islamabad Campus, Islamabad, Pakistan; ^5^Discipline of Clinical Pharmacy, School of Pharmaceutical Science, University Sains Malaysia, Gelugor, Penang, Malaysia

**Keywords:** pharmacy practice, lower-middle-income countries, fixed dose combinations, community pharmacy, national TB control program, multi drug resistant tuberculosis, infectious disease

## Abstract

**Introduction:**

The emergence of MDR-TB is a global threat and an obstacle to the effective control of TB in Pakistan. A lack of proper TB knowledge among the staff in private pharmacies and the sale of compromised quality anti-TB drugs are the main instigators of multidrug-resistant tuberculosis (MDR-TB). Thus, this study was aimed at investigating the quality and storage conditions of fixed-dose combination (FDC) anti-TB drugs along with the awareness of staff working in private pharmacies regarding the identification of potential patients with TB and dispensing the inappropriate treatment regimens contributing to MDR-TB.

**Methods:**

The study is completed in two phases. In phase I a cross-sectional study is performed using two quantitative research designs, i.e., exploratory and descriptive, to evaluate the knowledge of private pharmacy staff. The sample of 218 pharmacies was selected. While in phase II cross sectional survey is conducted in 10 facilities from where FDC anti TB drugs were sampled for analyzing their quality.

**Result:**

Results revealed the presence of pharmacists only at 11.5% of pharmacies. Approximately 81% of staff at pharmacies had no awareness of MDR-TB, while 89% of pharmacies had no TB-related informative materials. The staff identified that most of the patients with TB (70%) were of poor socio-economic class, which restricted their purchase of four FDCs only up to 2–3 months. Only 23% were acquainted with the Pakistan National TB Program (NTP). Except for MDR-TB, the results showed a significant correlation between the experiences of staff with TB awareness. Findings from the quality evaluation of four FDC-TB drugs indicated that the dissolution and content assay of rifampicin were not according to the specifications, and overall, 30% of samples failed to comply with specifications. However, the other quality attributes were within the limits.

**Conclusion:**

In light of the data, it can be concluded that private pharmacies could be crucial to the effective management of NTP through the timely identification of patients with TB, appropriate disease and therapy-related education and counseling, and proper storage and stock maintenance.

## Introduction

Drug-resistant tuberculosis (DR-TB) continues to be a public health threat around the globe (1). DR-TB cases can be classified on the basis of different resistance patterns to anti-TB drugs: monoresistance (resistance to one first-line anti-TB drug only), polyresistance (resistance to two or more of these drugs, except for both rifampicin and isoniazid), multiresistance (resistance to at least both rifampicin and isoniazid), and extensive resistance (resistance to both rifampicin and isoniazid, a fluoroquinolone, and at least one of the three second-line injectable drugs). Although the TB incidence rate is annually declining by 2% and an estimated 54 million deaths have been averted during the last 17 years, the increasing incidence and spread of DR-TB are putting these achievements at stake (2). Presently, a median of 9.9% of *Mycobacterium tuberculosis* strains is now resistant to at least one drug in 35 countries or regions (3). Worldwide, in 2014, 5% of MDR-TB cases were reported to the WHO, including 35% of new cases, while 75% of relapse TB cases were reported in Central Asian and eastern European countries (4).

Pakistan ranks fifth among the list of 30 high-TB burden countries in the world, with an estimated number of 5,18,000 TB cases, including 15,000 MDR-TB cases (5). The Ministry of Health Pakistan, considering the WHO's global emergency declaration, launched a national TB program (NTP) in 1995 against TB and implemented directly observed treatment Directly Observed Therapy Shortcourse (DOTS) with fixed-dose combinations (FDCs) tablets [i.e., isoniazid, (H) rifampicin, (R) ethambutol, (E) and pyrazinamide (Z) (HRZE)] as the first-line therapy in 2001 (6). This policy was followed by launching a Programmatic Management of Drug-Resistant Tuberculosis (PMDT) model of care in 2010 (7) as well as engaging private TB stakeholders on a large scale in 2014, where presumptive patients with TB initially seek respiratory healthcare (8). After launching both PMDT and FDCs strategies, an important objective of NTP was also set to produce a quality product (FDC) locally, meeting the international quality attributes. Although the NTP DOTS program resulted in a high rate of detection (84%), later on, therapy failures owing to the emergence of multidrug resistance tuberculosis (MDR-TB) were reported (9). There is considerable agreement that inadequate treatment or improper use of anti-tuberculosis medications contributed significantly to these therapy failures. Many studies have highlighted that non-adherence to the treatment regimen is due to the lack of knowledge on practices regarding TB, socio-economic barriers, and the attitude of patients and prescribers (10). In terms of information on standard TB treatment protocols, poor/inadequate knowledge among pharmacy technicians and pharmacists working at privately owned pharmacies with regard to differences between simple cough and TB symptoms can be another factor that contributes toward the emergence of MDR-TB due to management fumble. These factors raised the importance of including these privately owned pharmacies in National Tuberculosis Control Programs (NTPs) and training their staff about the importance of the program, highlighting their vital role in the control and proper management of TB (11). The engagement of community pharmacies in detecting TB cases has been briefly adopted and launched across three districts, but the quality of anti-TB medication sold to patients undergoing self-medication has not been explored yet (12). Also, a large network of private pharmacies exists who are working outside the umbrella of NTP and selling TB medications provided to them by medicine distribution/marketing teams without having any formal training and consultation in checking the quality of medicines procured by them (13).

Therefore, the present study was designed to investigate the quality and storage conditions of fixed-dose combination (FDC) anti-TB drugs sold at retail pharmacies and those under the supervision of National Tuberculosis Programs (NTPs). In addition, the knowledge of private pharmacy-associated staff was also monitored in terms of dispensing inappropriate treatment regimens without patients counseling, resulting in increased MDR-TB in the community of Pakistan.

## Methodology

The study was completed in two different phases.

### Phase I

A cross-sectional study was performed using two quantitative research designs, i.e., exploratory and descriptive, to evaluate the knowledge of private pharmacy staff. With an exploratory approach, the involvement of community pharmacies in “TB treatment outcome and control by NTP of Pakistan” was observed, while the descriptive study was designed to highlight the current practice and knowledge of community pharmacy staff and associated problems. Phase I was conducted in twin cities, i.e., Rawalpindi and Islamabad. The study period was 6 months.

### Phase II

Phase II was aimed at mapping the quality of anti-TB drugs. A cross-sectional survey was conducted in purposively chosen 10 facilities from where FDC anti-TB drugs were sampled. These collected samples were analyzed for dissolution, disintegration, friability, weight variation, and assay and compared with the United State Pharmacopeia (USP) standards. Phase II was conducted in two cities, i.e., Multan and Islamabad. These two cities were chosen purposively due to climatic differences. The temperature of Multan is relatively high with less humidity as compared to Islamabad having humid and low temperatures. Dry, well-lit, ventilated places at a temperature of 15–30°C and relative humidity of 60% are normal storage requirements for medicines.

#### Study center

A total of 208 pharmacies were included in the Phase I survey from both Rawalpindi and Islamabad. A purposive sampling technique was used to ascertain the pharmacies having staff with relevant working experience and duration. In Phase II, a minimum of 50 tablets from each pharmacy were collected for quality control tests. A total of 3,248 tablets were collected and analyzed for different quality control parameters.

The pharmacies were included in the study based on the following inclusion and exclusion criteria.

##### Inclusion criteria

All pharmacies (category A), or community retail outlets, having staff with a work experience of more than 7 years and willing to participate in the study were included in the study.

For Phase II, all TB medicines, manufactured by local pharmaceutical companies, with intact packing and under proper storage conditions being consumed by the patients with TB visiting health facilities under the NTP and private community pharmacies and willing to provide the sample were included for random sampling of the medicines.

##### Exclusion criteria

All retail community pharmacies having staff with a work experience of fewer than 7 years and not willing to participate in the study were excluded from the study.

For Phase II, any anti-TB drug with broken seal packing, expired, or not protected from excessive light, and with no consent for sampling by the NTP or by local manufacturers were excluded from Phase II.

#### Data collection

For Phase I, the standardized questionnaire tool (pre-tested) was used to collect the data from community pharmacy staff, and pre-test participants were excluded from the main study. All the data were collected through face-to-face interviews conducted at each pharmacy during working hours.

For Phase II, the FDCs containing rifampicin 150 mg, pyrazinamide 400 mg, isoniazid 75 mg, and ethambutol 275 mg in single tablets were sampled from two different facilities, i.e., retail pharmacies and public health facilities of Islamabad. Tablets sampled from retail pharmacies in Islamabad are coded as I, II, and III and those from Multan are coded as IV, V, and VI. Tablets sampled from two public health facilities in Islamabad were coded as W and X and those from Multan were coded as Y and Z. A total of 3,248 tablets were sampled from 10 facilities as shown in Table 1. The manufacturers of the sampled FDC tablets were coded as A, B, C, D, and E along with the percentage of tablets sampled.

**Table 1:** Selected facilities and number of samples collected from each facility in different cities of Pakistan.

**Selected facilities**	**Name of cities**	**Sample quantity**	**Number of tablets**
Retail pharmacy I	Islamabad	5 × 20	100
Retail pharmacy II		10 × 8	80
Retail pharmacy III		10 × 10	100
Retail pharmacy IV	Multan	10 × 8	80
Retail pharmacy V		5 × 20	100
Retail pharmacy VI		10 × 10	100
Public health facility W^*^	Islamabad	24 × 28	672
Public health facility X^*^		24 × 28	672
Public health facility Y^*^	Multan	24 × 28	672
Public health facility Z^*^		24 × 28	672
Total number of tablets sampled from 10 facilities			3248

^*^FDC-TB drugs dispensed under the supervision of NTP.

#### Data collection and quality control methodology

For the Phase II study, an observation checklist was used to collect the details of the sample, its storage conditions, and the place of collection. The quality control test of sampled FDCs was conducted following the procedure mentioned in the respective USP monograph using a weighing balance (OHAUS corporation, PA 214C, USA) for the weight variation test, dissolution apparatus *II* (Erweka, DT-820, Heusenstamm, Germany) for dissolution, and disintegration apparatus (Pharma Test, Germany) for disintegration time. High performance liquid chromatography (HPLC) (Shimadzu SPD 20A, Japan) for drug quantification in various samples.

#### Statistical analysis

Data were analyzed using Statistical Package for Social Sciences software version 22.0 (SPSS). The demographic profile of study contributors was entered as categorical variables. The knowledge and practice numbers of study contributors were added as continuous variables. According to bloom's cutoff points, the numbers were then categorized: 50% and below as poor, 60% and above as fair, and 70% and above as good (11).

In evaluating the potential of private pharmacies (Phase I), the frequency distributions of categorical variables, socio-demographic data, and practice, attitude, and knowledge of community pharmacy staff were estimated using a cross tab, while mean and median were estimated for continuous variables. The correlation between independent and outcome variables was determined using the Pearson chi-square test. However, *P*-value result was reported as statistically significant. While mapping the quality of the anti-TB drug (Phase II) study, data were entered into a Microsoft Excel sheet and then analyzed. A *p* ≤ 0.05 was considered statistically significant.

## Results

A total of 208 pharmacies were selected based on the willingness of pharmacy owners. The attributes of working staff and selected features of these pharmacies are shown in Table 2.

**Table 2:** Basic characteristics of working staff and selected pharmacies.

**Characteristics**	**Number (*n* = 200)**	**Percentage**
**Gender**		
Male	197	98.5
Female	3	1.5
**Age (years)**		
≤ 20	30	15.0
21–30	119	59.5
31–40	40	20.0
>40	11	5.5
**Staff category**		
Pharmacist	23	11.5
Pharmacy attendants	50	25.0
Salesman	127	63.5
**Experience (years)**		
≤ 5	153	76.5
6–15	21	10.5
>15	26	13.0
**Years of establishment**		
0–10	142	71.0
11–20	34	17.0
>20	24	12
**Clients per day**		
≤ 50	67	33.5
51–100	56	28.0
101–>200	77	38.5
**Client documentation**		
No documentation	132	66.0
Medicines sale register	64	32.0
Prescription filled	4	2.0
**TB information**		
No	178	89
Yes	22	11
**Working staff**		
1	23	11.5
2	63	31.5
3	39	19.5
4	17	8.5
5	15	7.5
>5	43	21.5

n, No of staff available at pharmacies.

### Basic characteristics of pharmacies and their working staff

The basic characteristics of the working staff and the selected pharmacies are summarized in Table 2. It was observed that among the working staff, most of the subjects were men (98.5%) aged between 21 and 40 years (79.5%) working as salesmen (63.5%) and had a working experience of ≤ 5 (76.5%) years (Table 2). As shown in Table 2, most of the selected pharmacies had a working life between 0 and 10 years (71%), clientage per day between 50 and 200 (66.5%), with minimum client documentation (32%), and dissemination of TB information (11%), and almost >60% for pharmacies having a staff range of 1–3 (Table 2).

### Awareness among working staff

Tuberculosis awareness among the pharmacy staff was evaluated on five parameters. Only 23% of the participants knew about the ntp, while 77% of staff were unaware of the existence of ntp, which is an alarming situation. The (participants) knowledge about the signs and symptoms of TB showed that most patients mentioned cough (more than 2 weeks) and then a tightness in breathing and high temperature at night which is 29, 14, and 13%, respectively, as some of the major signs of positive TB case. About the proliferation of TB in a community, 26% of participants responded that TB proliferation in a community is *via* poverty, 24% reasoned the existence of patients with TB for proliferation, while 21% have no information. For TB identification, 29% of pharmacy staff answered that the most frequent test performed is the sputum smear test, which is followed by the chest X-ray indicated by 18% of staff, while 13% had no idea about the diagnostic tests for TB. Data of the participants views on the possible control of TB showed that the majority (42%) of the staff had an idea that using a mask can inhibit TB spread, whereas 20% said that treatment can inhibit the TB spread and the use of a mask, while 16% had no information. Regarding the emergence of MDR, out of 200 pharmacies, 81% had no knowledge or heard about MDR-TB. Furthermore, when asked about factors contributing to MDR-TB, only 2% of pharmacy staff knew that it was inappropriate treatment and 11% said that the incompletion of recommended therapy is the reason behind that.

### Sale of anti-TB medicines

**Figure 1:**
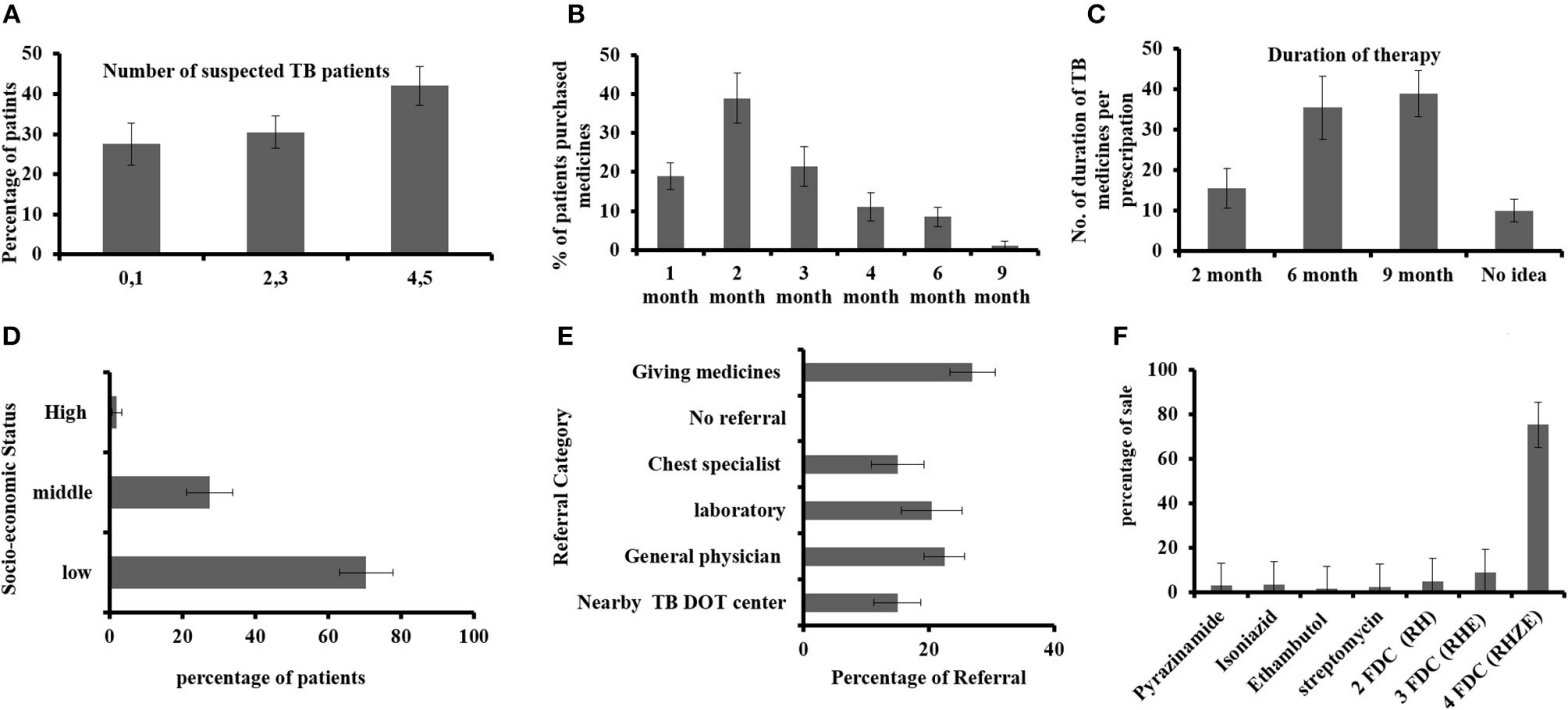
**(A)** Identification of suspected patients with TB per week, **(B)** duration of TB medicine purchased by the patients with TB, **(C)** TB medicine course interval, **(D)** socio-economic status of patients with TB, **(E)** physician referral of suspected patients, and **(F)** mostly sold anti-TB drugs.

The sale of anti-TB drugs was assessed through different parameters (as summarized in Figures 1A–F). Regarding the period of TB medicine purchase, almost 60% of interviewed staff answered that patients purchased TB medicines just up to 2 months (Figure 1B) only. When asked about the course of therapy, most of the participants (70%), as shown in Figure 1C, knew that TB therapy takes a 6-month time period, while 10% were unaware of that. The data showed that frequently sold anti-TB medicines were the four fixed-dose combination medicines, and to some extent, two (HZ: isoniazid, pyrazinamide) and three FDC (HZR: isoniazid, pyrazinamide, rifampicin) anti-TB drugs. Figure 1E shows the referral practice of suspected persons in which only 15% were referred to DOT centers, while 27% had no referral and they are giving medicines such as simple cough syrup and broad-spectrum antibiotics.

### Correlation of respondent's professional background with the awareness of TB

**Figure 2:**
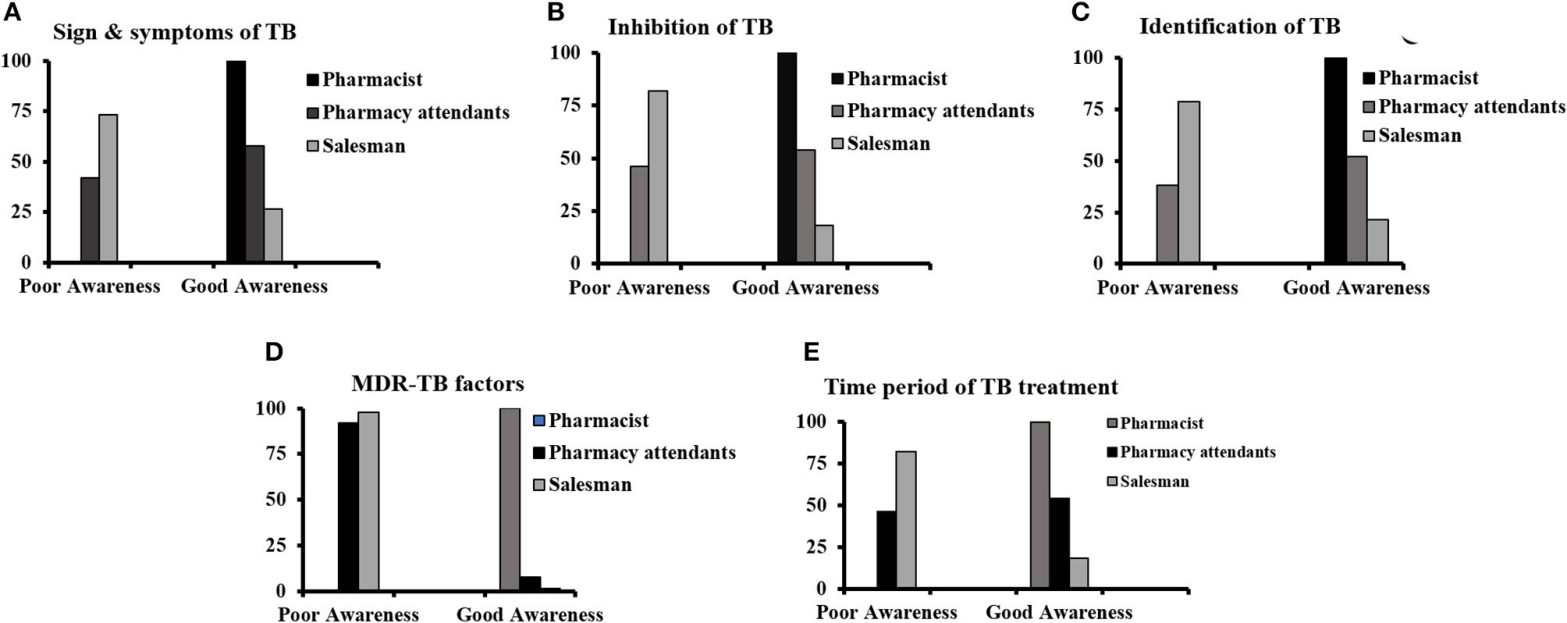
Correlation of respondent's professional background with the awareness of TB regarding **(A)** signs and symptoms, **(B)** inhibition of TB, **(C)** identification of patients with TB, **(D)** multidrug-resistant TB factors, and **(E)** time period of TB treatment.

The correlation between categories of working staff at private retail pharmacies and TB awareness is summarized in Figures 2A–E. In this study, TB awareness was significantly correlated with the professional background of participants and the *p*-value of 0.000 in all figures shows very strong evidence.

### Correlation of respondent's working experience in pharmacies with TB awareness

**Figure 3:**
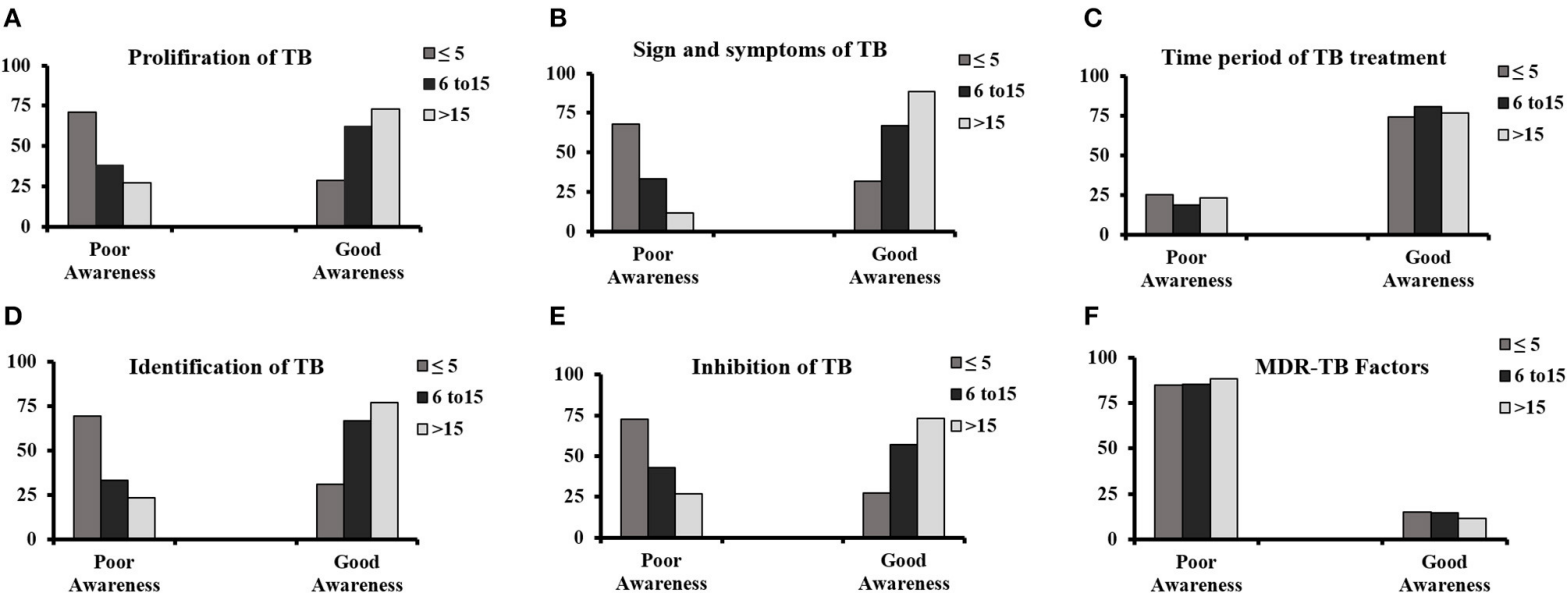
Correlation of respondent's working experience in pharmacies with TB awareness regarding **(A)** proliferation of TB, **(B)** signs and symptoms, **(C)** time period of TB treatment, **(D)** identification of patients with TB, **(E)** inhibition of TB, and **(F)** multidrug-resistant TB factors.

The correlation between working experience of respondents with TB awareness is summarized in Figures 3A–F. The study showed a direct relationship between TB awareness and the experience of staff. The participants with < 5 years' experience had poor knowledge in comparison with those with 6–15 and 15 years of experience. The awareness of TB was significant with a *p*-value of 0.000 and reveals a positive association with participants having work experience except for the MDR-TB factors with a *p* > 0.005.

### Correlation of respondents' work experience with the identification of patients with TB and the number of working staff with a load of clients

The results are summarized in Table 3. Pharmacies with a high number of working staff had a high number of clients' load, and similarly, pharmacies with a high number of clients' load had a high number of suspected TB persons. With the exception of respondents' work experience to the customer load, all other variables show a significant result.

**Table 3:** Number of patients with TB per week with regard to the number of staff and their working experience.

**Number of patients with TB per week**					
**No. of customer/day**	**Identification of patients with TB**			**Total**	***p*** **-value**
	**No. of patients with TB identified**				
	**0–1 (%)**	**2–3 (%)**	**4–5 (%)**		
≤ 50	32 (42.7)	27 (36.0)	16 (21.3)	75	0.0001^**^
51–100	14 (27.5)	18 (35.3)	19 (37.3)	51	
101–>200	9 (12.2)	16 (21.6)	49 (66.2)	74	
	**Staff experience/year**				
	≤ **5 (%)**	**6–15 (%)**	>**15 (%)**	**Total**	
≤ 50	58 (86.6)	6 (9.0)	3 (4.5)	67	0.114
51–100	40 (71.4)	7 (12.5)	9 (16.1)	56	
101–>200	55 (71.4)	8 (10.4)	14 (18.2)	77	
	**No. of pharmacy staff**				
	**1–2 (%)**	**3–4 (%)**	≥**5 (%)**	**Total**	
≤ 50	14 (20.9)	30 (44.8)	23 (34.3)	67	0.002^**^
51–100	6 (10.7)	25 (44.6)	25 (44.6)	56	
101–>200	3 (3.9)	25 (32.5)	49 (63.6)	77	

### Qualitative evaluation of FDCs in different places

The quality of medicines is the prime factor for the efficient treatment and eradication of disease within the course of therapy. The provision of the substandard drug is also a contributor not only to treatment failure but also may result in the emergence of MDR against anti-TB drugs. The quality attributes of FDCs provided at private pharmacies and the NTP monitored at public healthcare facilities were evaluated on different parameters. The storage conditions and environment of the facilities were evaluated to determine the effect on these medicines.

### Weight variation test

The results of weight variation studies conducted on various sampled FDCs are summarized in Table 4. The average weight of FDCs tablets was found within an acceptable range of USP (2007)^1^ for weight variation, that is, for tablets weighing >324 mg, ±5% is the deviation limit.

**Table 4:** Uniformity of weight in FDCs tablets sampled from different facilities.

**Selected facility**	**No of tablets**	**Deviation of weight**	**Conclusion**
Retail pharmacy I	20	No variation	Within range
Retail pharmacy II	20	No variation	Within range
Retail pharmacy III	20	No variation	Within range
Retail pharmacy IV	20	No variation	Within range
Retail pharmacy V	20	No variation	Within range
Retail pharmacy VI	20	No variation	Within range
Public health facility W	20	No variation	Within range
Public health facility X	20	No variation	Within range
Public health facility Y	20	No variation	Within range
Public health facility Z	20	No variation	Within range

### Friability

Friability analysis of all the tablets sampled from different places showed that FDCs tablets were within an acceptable limit of USP, that is, < 1% (USP, 2007)^1^.

### Disintegration time test

**Figure 4:**
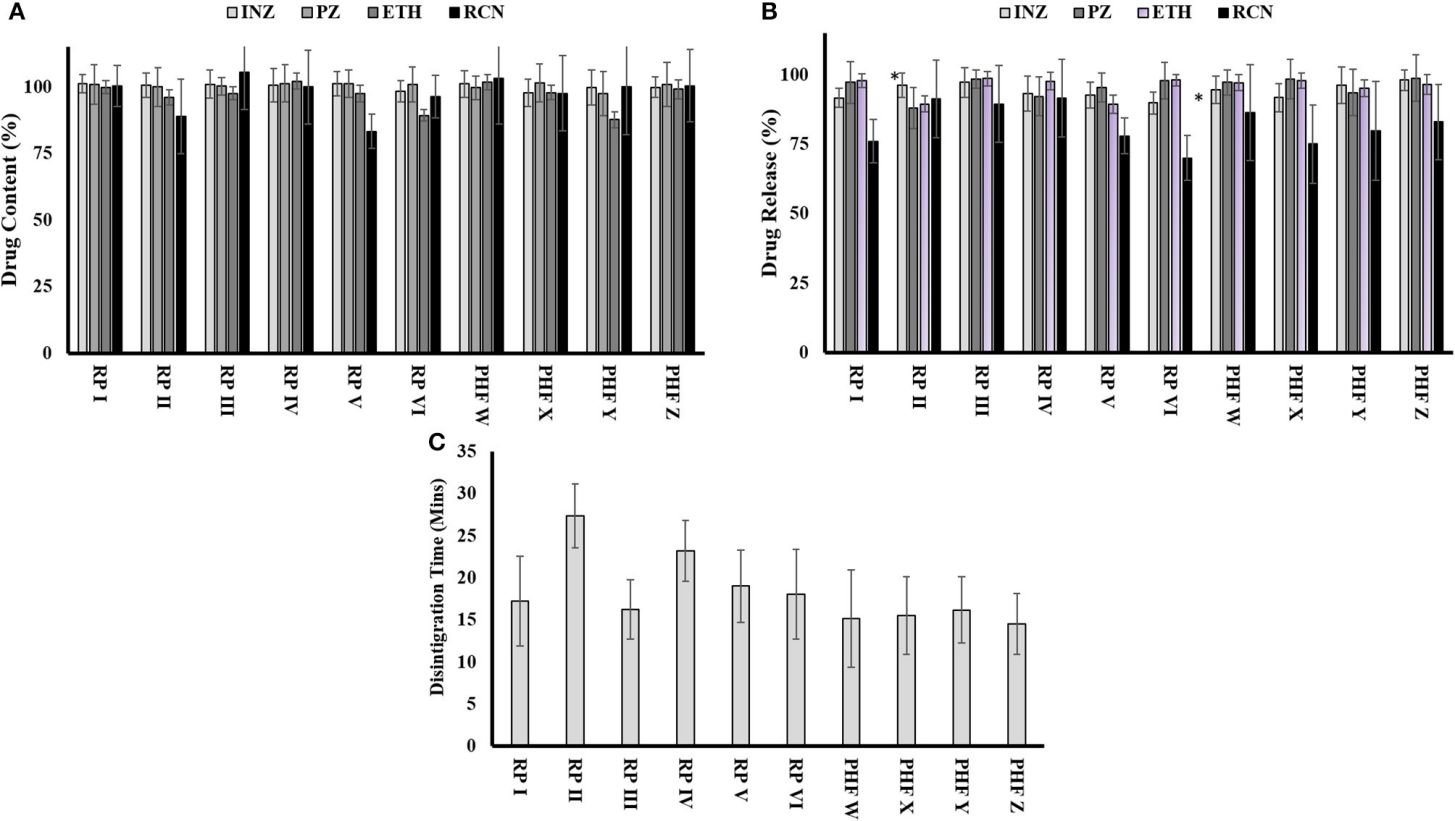
Quality attributes of FDCs samples collected from various places: **(A)** evaluation of content uniformity of drugs in 4-FDCs, **(B)** dissolution profile of drugs from 4-FDCs, and **(C)** disintegration time of 4-FDCs. ^*^RP, retail pharmacy; PHF, public health facility; INZ, isoniazid; PZ, pyrazinamide; ETH, ethambutol; RCN, rifampicin.

According to the standard procedure given in USP, the disintegration time for an uncoated tablet is < 30 min. The results of the disintegration time studied for sampled FDC tablets are shown in Figure 4C. These results indicated that FDCs tablet samples from different facilities disintegrated within 30 min and, thus, passed the test.

### Dissolution test

The result of the dissolution of isoniazid ranged from 91.6 for retail pharmacy I to 98% for public health facility Z. The dissolution of pyrazinamide indicated a minimum range of 92.2% at retail pharmacy IV to a maximum of 98.8% at public health facility Z. Drugs from all facilities complied with the dissolution of ethambutol and within limits, while in Figure 4B, the dissolution of rifampicin shows variation in one sample, which are 70% collected from retail pharmacy VI.

### Assay of different FDCs tablets sampled from different facilities

The limit of drug assay is between 90 and 110% of the mentioned label claim. The assay of isoniazid, pyrazinamide, and ethambutol was found within the acceptable limit of specifications given in USP for the samples collected from different places. The assay of rifampicin in FDCs tablets taken from facilities (retail pharmacies II and V) did not meet the assay limits, while the FDC tablets taken from the rest of the facilities were found within the USP acceptable limit. The results of the assay of each drug sampled from different facilities are shown in Figure 4A.

The quality control parameters evaluated for FDCs sampled from various places in both cities showed that overall 30% of the anti-TB FDCs sampled do not meet the specifications which might not produce the desired effects.

### Quality association between retail and public health facilities

The paired comparison *t*-test was applied at a level of significance of 5% (*p* = 0.05) in order to check the association in the quality attributes of FDCs sampled from different facilities. The results of FDC tablets in percent sampled from public health facilities (Islamabad and Multan) were taken as control as their results were within specifications, while the results of FDC tablets sampled from retail pharmacy facilities (Islamabad and Multan) were taken as test FDCs. Statistics showed that there is a significant association between control and test FDCs. The uniformity of weight, disintegration time, friability, dissolution, and the assay of pyrazinamide, isoniazid, and ethambutol from both retail and public health facilities were within specified limits; thus, for these tests, there was no quality difference between retail and public health facilities. However, in the case of dissolution studies of rifampicin between retail pharmacies and public health facilities, the *p*-value was 0.211, indicating non-significance. FDC tablets sampled from retail pharmacy facilities in the case of the content assay also showed non-significant association from the control as the *p-*values obtained for the assay was 0.0917. Overall, samples from three out of six retail pharmacies showed variations from reference parameters, while all the samples from public health facilities were within a specified range. However, the *p*-value is 0.0924, which is not a significant difference. This analysis suggests that *p*-values are greater than the level of significance ( ≤ 0.05) for the test parameters, indicating no significant association in the previously tested parameter when public health facilities in both regions were compared with test retail pharmacies.

### The followed practice of storage conditions of anti-TB FDC among selected facilities

The storage conditions of drugs from selected facilities were recorded based on an observation checklist. Table 5 shows FDC tablet storage conditions in private retail pharmacies and public health facilities. The packaging of all the samples was PVC-aluminum strip packs. Except for 1, all the premises were well-lighted and ventilated. The same situation was faced in shelving for drug storage. Among 10 facilities, eight used pallets at least 10 cm off the floor, while two used < 10 cm off the floor plates. No expired, damaged, or defective seals or drugs in all facilities were found. In all the selected facilities, there was no monitoring of humidity and temperature. The policy of the first in and first out was followed. None of the facilities had thermometer/temperature control cards. The results in Table 5 showed that there was no proper monitoring of humidity and temperature with the absence of thermometer/temperature control cards in every chosen facility. The table also showed that there was no proper ventilation system. So the excessive temperature and humidity may have affected the quality and efficacy of FDC drugs.

**Table 5:** Storage conditions of FDC among selected facilities.

**Factor**	**Response**	**Retail pharmacies (*n* = 6)**	**Public health centers (*n* = 4)**	**Public health center (*n* = 10)**
		**Facilities (%)**	**Facilities (%)**	
Form of packaging	Strip pack	6 (100)	4 (100)	10 (100)
	Loosely packed	0 (0)	0 (0)	0 (0)
	Strongly closed bottle protected from light	0 (0)	0 (0)	0 (0)
Ventilation	Yes	6 (100)	3 (75)	9 (90)
	No	0 (0)	1 (25)	1 (10)
Shelving for drug storage	Yes	6 (100)	3 (75)	9 (90)
	No	0 (0)	1 (25)	1 (10)
Pallets used at least 10 cm off the floor	Yes	5 (83.33)	3 (75)	8 (80)
	No	1 (16.66)	1 (25)	2 (20)
Drugs with damage seals, defective packs, or expiry	Yes	0 (0)	0 (0)	0 (0)
	No	6 (100)	4 (100)	10 (100)
Inspect packages for expiry or defective medicines on receipt	Yes	6 (100)	4 (100)	10 (100)
	No	0 (0)	0 (0)	0 (0)
Drugs stored on floor	Yes	0 (0)	1 (25)	1 (10)
	No	6 (100)	3 (85)	9 (90)
Humidity and temperature monitored	Yes	0 (0)	0 (0)	0 (0)
	No	6 (100)	4 (100)	10 (100)
Adequate storage space	Yes	6 (100)	4 (100)	10 (100)
	No	0 (0)	0 (0)	0 (0)
First in first out practice	Yes	6 (100)	4 (100)	10 (100)
	No	0 (0)	0 (0)	0 (0)
Temperature control cards/thermometer present	Yes	0 (0)	0 (0)	0 (0)
	No	6 (100)	4 (100)	10 (100)
First expiry first out practice	Yes	6 (100)	4 (100)	10 (100)
	No	0 (0)	0 (0)	0 (0)

## Discussion

The failure in TB management and emergence of MDR or XDR TB is an outcome of various underlying factors such as timely diagnosis, the provision of quality medications along with proper counseling, and most importantly, the involvement and commitment of all stakeholders to decrease the TB burden. The current research was designed to explore two factors, i.e., the evaluation of the privately owned retail pharmacies as a neglecting link in TB control in terms of TB awareness/knowledge among pharmacy staff. Second, the determination of the quality of four FDC anti-TB drugs available at retail pharmacies in comparison to public health centers provided by the NTP with their storage condition practice in the selected facilities.

One of the essential findings of our study was the lack of awareness about TB among private retail pharmacy-associated staff leading to unsatisfactory consultation and an inability to differentiate simple cough from TB symptoms. Among private retail pharmacies and staff, the same delusions and awareness gaps with respect to TB and its treatment were reported in several studies (12, 13). Pharmacy-associated staff with poor or no awareness about TB were unable to deal with and determine the patients with active TB. Proficient staff with TB knowledge elegantly establish a connection with patients with TB to expand TB knowledge in public and endorse adherence among patients, thus diminishing the rise of MDR-TB. The results highlighted a dire need for training the pharmacy staff with a healthcare framework and NTP regarding capacity building with directions on referral, signs and symptoms screening, and therapy support in TB control.

Another important finding of our study was the lack of qualified personnel in private retail pharmacies as depicted in Table 2 which is also supported by other similar research (14, 15). Qualified pharmacists in Pakistan do not prefer working at private pharmacies, and only 10% of pharmacists work in pharmacies (16). Low-income nations have a normal of 1.2% professional pharmacists for each 10,000 of the population in comparison with 4.4% in middle-income and 10.8 in high-income nations (17). The community sector of Pakistan can be helpful for TB control. Pharmacists and well-established community pharmacies can play a vital role in TB prevention and treatment. Most patients with TB do not follow their recommended course of therapy and directions associated with their disease leading to the emergence of drug resistance. Therefore, proper counseling for adherence to the prescribed therapy is essential for completing the course of therapy. Thus, for improving patient compliance and the rational use of medicines, there must be a qualified pharmacist in pharmacies at each level. Our research findings showed that most of the pharmacies dispensed simple cough and broad-spectrum antibiotics to patients with cough and fever symptoms, which lead to delayed TB diagnosis and resistance development (Figure 2E). The development of drug-resistant TB and TB diagnostic delays has been directly associated with the overuse of fluoroquinolones (18).

The patients buying anti-TB drugs from private retail pharmacies, with or without a prescription, showed less adherence and were less likely to complete the recommended course of therapy. In this study, almost 75% of patients purchased anti-TB medicine only for a 2–3-month duration which clearly indicates poor adherence to the required treatment (Figure 2B). The lack of collaboration between pharmacies and public-sector TB control authorities may mean that TB medicines are unwittingly sold without knowledge about their rational use. The majority of patients with TB from the lower socioeconomic class visited private retail pharmacies and purchased TB medications for only a 7-day therapy at a time. Earlier studies also reported the same results (19, 20). Urbanization-associated factors such as poverty and other social forces made the patients with TB less adherent to complete the required course of treatment, thus, it might be a contributing factor to the emergence of drug resistance in the community of Pakistan. This study found that 89% of pharmacies had no informative stuff regarding TB (Table 2). The studies showed variations in the identification of TB suspected persons, i.e., 1–3 persons per week.

In different studies in India, successful collaboration between Regional National TB Control Program (RNTCP) and private sectors has been accredited to improve the case detection rates of patients with TB (21). Our study indicated that the majority of dispensing drugs in private retail pharmacies were the four fixed-dose combination anti-tuberculosis drugs (Figure 3F). The use of poor-quality drugs is also one of the contributing factors to drug resistance in healthcare sectors. Thus, the second phase of the study aimed to evaluate the quality of anti-TB drugs, i.e., four FDCs (isoniazid, pyrazinamide, rifampicin, and ethambutol) provided to patients. The tablets were sampled from 10 different facilities (six retail pharmacies and four public health facilities) in two different climatic cities, i.e., Islamabad and Multan, Pakistan. Physical characteristics such as weight variation, friability, disintegration test, and content uniformity assay were performed in accordance with official procedures and specifications. All the physical characteristics tests for all facilities were found within the official limits and with no variations. But in case of chemical characteristics, rifampicin failed assay content test i.e., one from Multan and one from Islamabad. FDCs sampled from one retail pharmacy failed a dissolution test for rifampicin which was from Multan (Figure 4B). There was no significant variation among the different facilities, but overall, 30% variations were found. The variations might be due to defective manufacturing or due to the instability of rifampicin in combination with the other drugs present in the product due to the climatic effect. Different researchers have already reported variations in the physio-chemical properties of anti-TB FDCs products, and the results of present studies are a confirmation of what is reported earlier. Previously, the physicochemical stability of different FDCs (rifampicin, isoniazid, pyrazinamide, and ethambutol) available in India under ICH (48°C with RH of 75%) and WHO guidelines were conducted for a period of 3 months with/without packaging. The results showed the content of rifampicin, isoniazid, and pyrazinamide ranging from 90 to 110% initially, which after exposure to accelerated climatic conditions exhibited severe physicochemical instability for an unpackaged and packaged product. The possible reason behind instabilities might be the pyrazinamide with ethambutol that played a catalytic role in the isoniazid and rifampicin interaction (22).

The quality features of FDC and single drug preparations from different pharmacies and TB program centers manufactured by different countries (Estonia, Colombia, Latvia, India, and Vietnam) have already been evaluated, and results have described the effect of storage conditions on these medicines which somehow compromised the quality of FDCs (23).

This study revealed the presence of drugs with poor quality at private retail pharmacies, particularly in terms of dissolution and low content of rifampicin, one of the important antibiotics in TB treatment. Poor efficacy or low-quality drugs is an important factor in treatment failure and the emergence of resistance against anti-TB drugs. Thus, strict compliance with the storage conditions must be observed to protect the drug contents in anti-TB drugs in the market. Private pharmacies and TB regulatory authorities' collaboration is important as a combined effort to offer better care to patients with TB and avoid drug resistance in Pakistan. Pharmacoeconomics is a significant factor that governs compliance with long-term therapy. It was noticed that due to financial problems, most patients discontinue their treatment which is one of the major factors in the emergence of drug resistance. Another important observation was the stigma associated with TB, which possibly leads to most of the patients buying medicine from private pharmacies rather than NTP, which provides free diagnostic and treatment services (24, 25). Significant improvements were seen in the notification of TB cases and therapy completion ratio after the contractual engagements of pharmacy owners in South Asia (26, 27), which indicates that the proper involvement of private pharmacies in the TB control program can make a significant difference in the disease burden and decrease the MDR. Thus, this study suggests that to implement the laws, there should be the presence of at least one certified pharmacist in each and every pharmacy. In addition, the recognition of community pharmacy-associated staff as a major and necessary healthcare provider for communicable and chronic diseases is important. The NTP should continue private pharmacy staff training and support to encourage them to take part in TB control. There is a need for public awareness to provide confidence to citizens that demand appropriate counseling regarding their disease and the medication use of TB from private pharmacy staff.

## Conclusion

The study highlighted that the overall qualifications and training of technicians working at community pharmacies in Pakistan are inadequate regarding TB management. Patient adherence to TB medication is much higher for public health facilities as they provide free of cost treatment. The storage conditions were satisfactory to some extent in all selected private pharmacies and public health facilities, with an exception for temperature and humidity, which were not monitored in any of the selected facilities. The storage conditions showed some effects on the quality of the FDCs as 30% of samples showed variations from specifications for rifampicin content and dissolution profile as they failed to comply with the specifications. Moreover, the study also highlighted the implementation of the laws ensuring the presence of at least one certified pharmacist for proper supervision and ensuring the quality of medicine with proper storage conditions. The involvement of privately owned pharmacies and pharmacists in the TB control program can make a significant difference in terms of proper medication provision with patient counseling ensuring maximum adherence to the course of therapy.

## Data availability statement

The original contributions presented in the study are included in the article/supplementary material, further inquiries can be directed to the corresponding author.

## Ethics statement

Ethical approval for conducting this project was obtained from the Advanced Studies and Research Board, Quaid-i-Azam University, Islamabad (REC-RIPS-2020/443).

## Author contributions

FB conceived and designed the study, collected, interpreted, analyzed the data, drafted the first version of the manuscript, and revised the final manuscript. MS assisted in the data collection and reviewed the first draft. HH coordinated in data collection and revising of the manuscript. WU assisted in the analysis and writing. AK provided advice on data analysis and interpretation. GS assisted in the conceptual phase and study design, reviewed the first draft, and provided approval for the final manuscript. All authors contributed to the article and approved the submitted version.

## Footnotes

^1^ https://www.usp.org/sites/default/files/usp/document/harmonization/gen-chapter/g06_pf_ira_32_2_2006.pdf
